# Preparation and antimicrobial evaluation of polyion complex (PIC) nanoparticles loaded with polymyxin B

**DOI:** 10.1016/j.eurpolymj.2016.08.023

**Published:** 2017-02

**Authors:** Ignacio Insua, Sieta Majok, Anna F.A. Peacock, Anne Marie Krachler, Francisco Fernandez-Trillo

**Affiliations:** a School of Chemistry, University of Birmingham, B15 2TT Birmingham, UK; b School of Biosciences, University of Birmingham, B15 2TT Birmingham, UK; c Institute of Microbiology and Infection, University of Birmingham, B15 2TT Birmingham, UK

**Keywords:** PIC particles, Polyelectrolyte complexes, Antimicrobials, Drug delivery

## Abstract

Here, we describe novel polyion complex (PIC) particles for the delivery of Polymyxin B (Pol-B), an antimicrobial peptide currently used in the clinic as a last resort antibiotic against multidrug-resistant gram-negative bacteria. A range of conditions for the controlled assembly of Pol-B with poly(styrene sulphonate) (PSS) has been identified which let us prepare stable colloidal PIC particles. This way, PIC particles containing different Pol-B:PSS ratios have been prepared and their stability under simulated physiological conditions (*i.e*. pH, osmotic pressure and temperature) characterised. Furthermore, preliminary evaluation of the antimicrobial activity of these Pol-B containing PIC particles has been performed, by monitoring their effect on the growth of *Pseudomonas aeruginosa*, an opportunistic gram-negative bacterium.

## Highlights

• We report the preparation of PIC particles loaded with clinically relevant antibiotic Polymyxin B.
• PIC particle formulation is optimised to accommodate different loadings of Polymyxin B.
• PIC particles inhibit the growth of *Pseudomonas aeruginosa* in a particle dependent manner.

## Graphical Abstract

**Figure:**
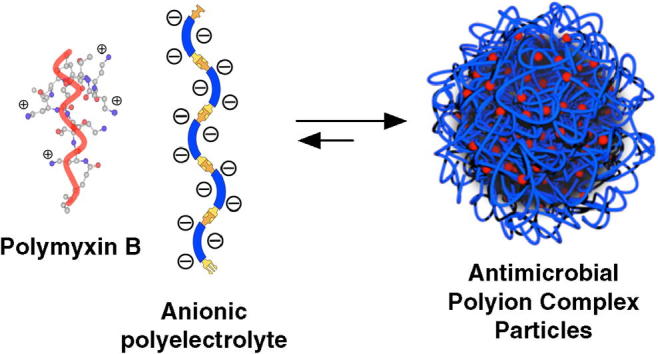

## 1 Introduction

The increasing number of drug-resistant bacteria poses a serious risk to global health [1], [2]. This increase in antimicrobial-resistant strains and the lack of new drugs reaching the market is encouraging the use of alternative approaches for the treatment of these infections [3], [4]. One of these strategies is the resurgence of “old” antibiotics such as polymyxins, which are now prescribed more often as last-resort antimicrobials against gram-negative multidrug-resistant strains [5], [6], [7]. However, polymyxins are toxic, and therefore strategies are required to minimise this toxicity and ensure that effective dosing of the antimicrobial is achieved [8]. Moreover, with the emergence of transmittable resistance to polymyxins [9], [10], strategies that can minimise systemic exposure to polymyxins are highly desirable.

Toxicity in polymyxins is associated with their amphiphilic and cationic nature. Therefore, the current strategy to minimise their toxicity is either to reduce the number of cationic residues and amphiphilic character [11], or to synthesise pro-drugs where cationic groups are masked to give a negatively-charged derivative [12], [13]. However, both strategies often result in a loss of antimicrobial activity, because this activity is directly linked to the presence of these cationic charges. The synthesis of polymer-drug conjugates has been employed as an alternative [14], [15], but this strategy still relies on the covalent attachment of polymyxin (or derivatives) to the polymer backbone, with the resulting loss in activity [14], [16].

**Fig. 1:**
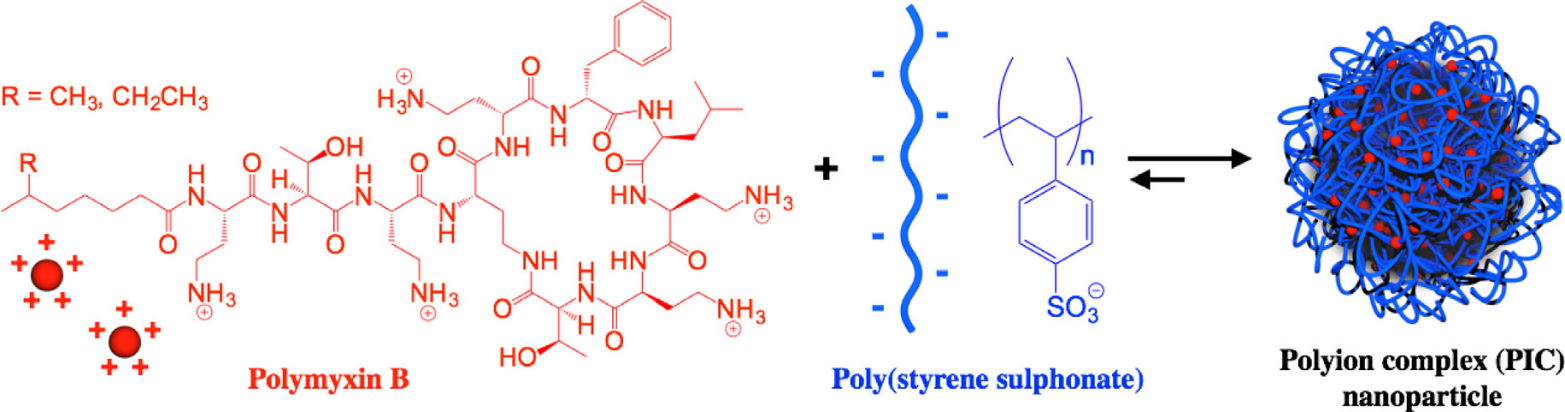
Assembly of PIC nanoparticles from cationic antimicrobial polymyxin B and anionic PSS.

There are several strategies for the delivery of antimicrobial peptides and derivatives [17], [18]. We and others have been exploring the use of polyion complex (PIC) nanoparticles as a platform for the delivery of this type of antimicrobials [19], [20], [21], [22], [23], [24]. In this approach, the positive charge in cationic antimicrobial polymers and peptides is neutralised in the presence of a negatively charged “polymer”. Under the right conditions, coacervate particles are formed upon neutralisation [25]. This way, positive charges are shielded from solution, without having to modify the structure of the antimicrobial (Fig. 1). In this article we describe the preparation of PIC nanoparticles as drug carriers for cationic polymyxin B sulphate (Pol-B), prepared by complexation of this antimicrobial with anionic poly(styrene sulphonate) (PSS). Different formulations were obtained by adjusting the Pol-B loading within these nanoparticles, and the formed PIC particles were characterised by Dynamic Light Scattering (DLS), ζ-potential and Transmission Electron Microscopy (TEM). Then, the stability of these nanomaterials was assessed under simulated physiological conditions. Finally, preliminary evaluation of the antimicrobial activity of these Pol-B:PSS PIC particles was done against *Pseudomonas aeruginosa*, an opportunistic gram-negative bacterium, and compared to that of free Pol-B to evaluate the differences in antibiotic activity of free and complexed drug.

## 2 Experimental

### 2.1 Materials

Polymyxin B sulphate (Pol-B) was purchased from Alfa Aesar®. Sodium poly(styrene sulphonate) 70 KDa average molecular weight (PSS), 4-(2-hydroxyethyl)piperazine-1-ethanesulfonic acid (HEPES), phosphotungstic acid hydrate and Luria Bertani (LB) broth (Miller) were bought from Sigma-Aldrich Co. Nylon 0.45 μm syringe filters were purchased from Camlab Ltd. Carbon-coated copper TEM grids (200 mesh) were purchased from Agar Scientific Ltd. Float-a-Lyzer® G2 (20 KDa MWCO) dialysis membranes were purchased from Spectrum Laboratories Inc. Dulbecco’s phosphate buffered saline (DPBS), 9.5 mM in phosphate and without Ca^2+^ and Mg^2+^, was bought from Lonza. Agar was purchased from Sigma-Aldrich Co.

### 2.2 Instrumentation

Dynamic light scattering (DLS) and ζ-potential measurements were carried out in a Zetasizer Nano ZS (Malvern Instruments Ltd.) stabilised at 37 °C. DLS was read at 173° (backscattering) for 60 s in triplicate and ζ-potentials were recorded 30 times at 140 V. TEM images of PIC nanoparticles were acquired on a JEM-1200EX (JEOL USA Inc.). PIC particle size was measured from TEM micrographs using ImageJ software (version 1.48v) and measuring each nanoparticle twice: both in their longest and shortest diameters. Reverse phase HPLC analysis was run through a Kinetex® C18-EVO column (Phenomenex®): 5 μm, 100 Å, 250 × 4.60 mm, stabilised at 35 °C and fitted to a SPD-M20A UV–vis detector (Shimadzu Co.) monitoring absorbance at 210 nm. A Loctite® LED flood array (Henkel Ltd.) operating at 405 nm was used to sterilise the samples studied in the bacterial growth experiment. A FLUOstar Omega (BMG Labtech GmbH.) microplate reader was used to incubate and measure the optical density at 600 nm (OD_600_) in the bacterial growth experiments. Pictures of agar plates were taken on a ChemiDoc™ MP imaging system (Bio-Rad laboratories Inc.).

### 2.3 Preparation of PIC nanoparticles

PIC particles from Pol-B and PSS were prepared following the protocol previously described by our group [20]. The different formulations of PIC particles are defined by their [N:SO_3_Na] ratio, which represents the proportion of amines in Pol-B over sulphonate groups in PSS. As an example, for the preparation of PIC particles at 0.5 [N:SO_3_Na] (*i.e.* half the amount of amines compared to sulphonate groups), solutions of Pol-B (0.25 mM) and PSS (2.5 mM in monomer units) were prepared separately in 5 mM HEPES buffer at pH 7.4. Then, both stock solutions were filtered through 0.45 μm nylon syringe filters and mixed in equal amounts drop-wise under stirring. PIC nanoparticles at other [N:SO_3_Na] ratios were obtained by changing the concentration of the Pol-B stock solution and mixing with 2.5 mM PSS following the same protocol. After 24 h, samples were characterised by DLS and ζ-potential without prior dilution nor filtration.

### 2.4 Stability of PIC nanoparticles in simulated physiological conditions

To a sample of PIC particles (1 mL), prepared as described above, 182 μL of a 1 M solution of NaCl in water was added and the mixture was incubated at 37 °C to obtain physiological osmotic pressure and temperature. Every hour, the sample was analysed by DLS as described above.

### 2.5 Pol-B release from PIC particles

2.5 mL of a PIC particle sample prepared at 0.7 [N:SO_3_Na] ratio were transferred into a pre-conditioned dialysis membrane (Float-a-Lyzer® G2), which was dialysed against 15 mL of DPBS at 37 °C under stirring (300 rpm). 500 μL of dialysate were taken at different time points and replaced with fresh DPBS. The dialysate samples were analysed by HPLC using an adaptation of the method previously reported by Orwa *et al.* [26], using a 50:23:22:5 mixture of 50 mM Na_2_SO_4_ in H_2_O: H_2_O: AcCN: 5% (v/v) dilution of 85% (m/m) H_3_PO_4_ in H_2_O running at 1 mL/min. 3 replicates were studied under the same conditions. At every time point, the area under the peak of Pol-B (*R*_t_ = 2.7 min) found in each dialysate sample was normalised to the area found in a 2.5 mL sample of Pol-B prepared at the same concentration as that contained in PIC particles at 0.7 [N:SO_3_Na] ratio (175 μM) diluted with 15 mL of DPBS (100% release). The same protocol was followed using a 175 μM Pol-B sample in 5 mM HEPES buffer pH 7.4 instead of PIC particles.

### 2.6 Growth curves of bacteria with PIC particles

50 μL aliquots of *P. aeruginosa* PAO1V cultures in LB broth (OD_600_ = 0.2) were loaded in the designated positions of a 96-well microplate. These bacterial cultures were mixed with 50 μL of PIC particles or free Pol-B in 5 mM HEPES buffer pH 7.4 to compare the antimicrobial activity of free and complexed (within PIC particles) Pol-B at the same concentration. In addition, these bacterial cultures were mixed with 50 μL of 5 mM HEPES buffer pH 7.4 as positive growth control and 1.25 mM (in monomer units) PSS in 5 mM HEPES buffer pH 7.4. Finally, 100 μL of a 1:1 mixture of the LB broth and 5 mM HEPES buffer pH 7.4 was also spotted in the microplate to confirm the sterility of the media. All samples were prepared in triplicate, with mean values and standard deviations reported. The microplate was incubated at 37 °C and orbital shaking, and the OD_600_ was monitored every 30 min for at least 15 h. The samples of PIC particles, free Pol-B, PSS and 5 mM HEPES buffer 7.4 were sterilised prior to this experiment by exposure to blue light (405 nm) for 2 h at a distance of 15 cm from the light source.

### 2.7 Antimicrobial assay of PIC particles: CFU counting

100 μL aliquots of a *P. aeruginosa* PAO1V culture in LB broth (OD_600_ = 0.2) were mixed with 100 μL of either PIC particles prepared at different [N:SO_3_Na] ratios or Pol-B solutions at the same concentration found in the PIC particles. In addition, the *P. aeruginosa* PAO1V culture was mixed likewise with 5 mM HEPES buffer pH 7.4 and a 1.25 mM PSS solution in this same buffer as controls. All mixtures were incubated at 37 °C for 22 h. After this time, 10 μL of each sample were spotted in triplicate on an agar plates in serial dilution, from undiluted down to a 10^8^-fold dilution in 10^2^ increments. These agar plates were then incubated at 37 °C. After 16 h of incubation, the agar plates were photographed and bacterial concentrations in CFU/mL were calculated from the highest dilution that showed individual colonies. All samples were sterilised prior to this experiment by exposure to blue light (405 nm) for 2 h at a distance of 15 cm from the light source.

## 3 Results and discussion

Our aim was to develop and evaluate a novel nanoparticle system for the delivery of Pol-B, easy to prepare, inexpensive and with tuneable antimicrobial loading capacity. Based on the pentacationic structure of Pol-B, we postulated the preparation of polymeric nanoparticles by incubating this antibiotic under mild conditions with a negatively charged polyelectrolyte. We anticipated that a strong polyelectrolyte such as PSS would facilitate complexation of Pol-B, and compensate for the relative low multivalency (*i.e*. number of cationic residues) of this antibiotic: Pol-B has five amine residues derived from diaminobutanoic acid (Fig. 1). At physiological pHs, these amines should be “fully” cationic with at least 95% protonation (*pK_aH_* 9–10) [27], giving Pol-B only five positive charges. Moreover, a considerably high molecular weight PSS (70 KDa) was selected to maximise the entropic gain upon PSS:Pol-B complexation. Also, PSS presents a hydrophobic styrene backbone, which could facilitate interactions with the hydrophobic residues in Pol-B and its aliphatic chain, that gives Pol-B an amphiphilic character (Fig. 1). Finally, it should be mentioned that PSS is an already approved pharmaceutical ingredient [28] and thus constituted an ideal starting point to develop our Pol-B containing PIC particles.

The preparation of Pol-B containing PIC nanoparticles was optimised by mixing stock solutions of this antimicrobial (in 5 mM HEPES buffer) with a 2.5 mM (per monomer) stock solution of PSS in the same buffer, following a protocol recently employed in our laboratories for the preparation of PIC particles containing short enzyme-responsive peptides [20]. At first, a broad range of [N:SO_3_Na] ratios was explored going from a 5-fold excess of cationic residues to a 5-fold excess of anionic groups (Fig. S1†). Not surprisingly, when equimolar amounts of ammonium and sulphonate groups were mixed, unstable suspensions were obtained that flocculated. The presence of these macroscopic aggregates suggested that neutral coacervates were being formed, which were not stable in suspension and eventually phase separated. The same result was obtained for all the formulations prepared using an excess of positive charge ([N:SO_3_Na] > 1) (Fig. S1†), suggesting that the sequential adsorption of Pol-B molecules on PSS neutralises the initial negative charge in the primary complexes formed [25]. Further addition of Pol-B once these neutral flocculates are formed is redundant, as they cannot be recruited into the forming particles due to the lack of negative charge in these aggregates. This behaviour has been observed in the literature during the formation of PIC particles from small oligomeric electrolytes [20], [23], [25]. Interestingly, flocculation was also observed in mixtures below but very close to equimolarity (0.8 [N:SO_3_Na] ratio) suggesting that not all styrene units had a sulphonate group. A commercial source of PSS was used in this work and the observed deviation from equimolarity could be explained because the industrial production of PSS from polystyrene results in different degrees of sulphonylation.

**Fig. 2:**
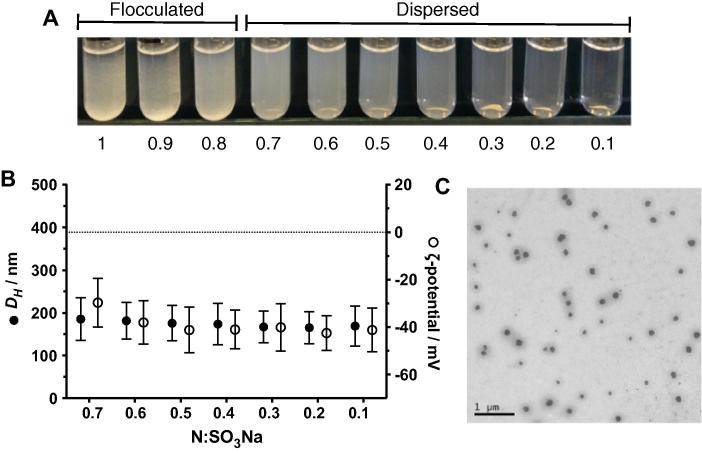
(A) Macroscopic appearance of particles prepared at different [N:SO_3_Na] ratios. (B) Hydrodynamic diameter (*D_H_*, ●) and ζ-potential (○) of PIC particles prepared at different [N:SO_3_Na] ratios. (C) Representative transmission electron micrograph of PIC particles prepared at a 0.4 [N:SO_3_Na] ratio; Scale bar = 1 μm.

Having established that colloidal particles were only obtained at sub-stoichiometric ratios of Pol-B, this range was again explored but 10 different formulations were investigated (Fig. 2A). Again, flocculation was observed for those formulations prepared with ratios close to equimolarity (1, 0.9 and 0.8 [N:SO_3_Na] ratios). Yet, we were able to obtain colloidal PIC particles from 0.7 to 0.1 [N:SO_3_Na] ratios, all of which showed very similar sizes, with mean hydrodynamic diameters between 166 and 186 nm (Fig. 2B and Table S1†). All PIC particles obtained were negatively charged (ζ-potential of *ca.* −40 mV), as expected the excess of PSS present in these formulations. Particles prepared at a 0.7 [N:SO_3_Na] ratio displayed a slightly smaller ζ-potential of −30 mV (Fig. 2B). While the difference in charge was small, this reduction in charge follows the expected trend towards less negatively charged particles as the formulation approaches equimolarity. TEM was performed on PIC particles prepared at a 0.4 [N:SO_3_Na] ratio, selected as a representative example of all seven formulations characterised by DLS (Fig. 2C and Fig. S2†). These images confirmed the spherical shape of these nanomaterials and an average particle diameter of 140 ± 30 nm (*n* = 58), slightly smaller than the hydrodynamic diameter measured by DLS (174 ± 49 nm). This apparent reduction in size found by TEM can be attributed to drying upon sample preparation, which could result in these highly hydrated nanomaterials shrinking as the solvent evaporates [29].

**Fig. 3:**
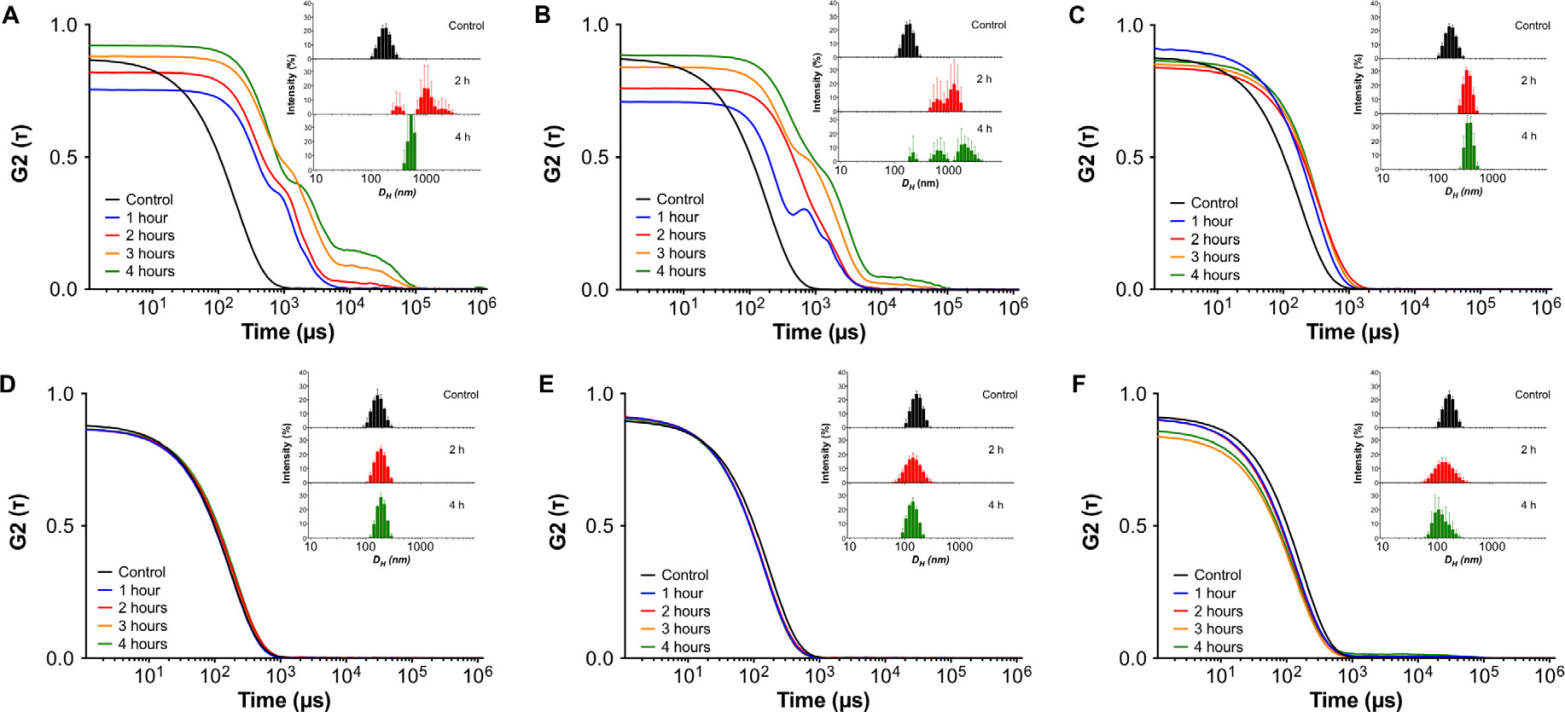
Autocorrelation function (ACF) curves and representative size-intensity distributions (inset) for PIC particles prepared using six different [N:SO_3_Na] ratios in the absence (control) and presence of 154 mM NaCl at 37 °C over time (1–4 h). 0.7 (A), 0.6 (B), 0.5 (C), 0.4 (D), 0.3 (E), 0.2 (F) [N:SO_3_Na] ratio. Because of the dispersion in DLS measurements observed for unstable particles (*i.e.* 0.1:1 [N:SO_3_Na] ratio) only representative plots are shown. 3 technical replicates were recorded for each sample.

Having identified a range of ratios from which Pol-B containing colloidal PIC particles could be prepared, the stability of these particles under simulated physiological conditions was assessed. It is well established that the presence of small electrolytes can shield the electrostatic attraction between the oppositely charged components of these nanomaterials. This screening normally results in particle swelling and can eventually lead to the disassembly of the PIC particle [25]. Since Pol-B loading dictated the number of electrostatic cross-links present in these colloids, it was anticipated that different [N:SO_3_Na] ratios would behave differently in the presence of biologically relevant salt concentrations. Thus, the seven different formulations of PIC particles identified so far were incubated with 154 mM NaCl at 37 °C and analysed over time by DLS, to assess their tolerance to physiological conditions (*i.e.* osmotic pressure and temperature) (Fig. 3). Broadly, particles behaved in three different ways when challenged with physiological conditions:

(1) A remarkable increase in particle size was observed for those PIC particles prepared at 0.7 and 0.6 [N:SO_3_Na] ratios (Fig. 3A and B). Micron-sized aggregates could be identified for these formulations, probably due to a combination of swelling and inter-particle aggregation as observed for other PIC particle systems [30].
(2) A region of high particle stability was found for formulations prepared between 0.5 and 0.2 [N:SO_3_Na] ratios, for which the mean size remained almost unchanged during this experiment. For this group, only particles prepared at a 0.5 [N:SO_3_Na] ratio changed their size significantly upon exposure to higher osmotic pressure, doubling their size after two hours. These particles then remained swollen and unchanged for the following hours (Fig. 3 and Fig. S4†).
(3) PIC particles prepared at a 0.1 [N:SO_3_Na] ratio displayed very poor stability under these conditions, breaking into small-sized amorphous aggregates, as suggested by the loss of a sigmoidal profile in their DLS autocorrelation function curves (Fig. S3†). Overall, PIC particles at 0.1 [N:SO_3_Na] exhibited the lowest tolerance to salt.

These stability experiments suggested that PIC particles could break apart in the presence of physiological conditions and therefore release the encapsulated Pol-B. To further demonstrate this release, PIC particles prepared at a 0.7 [N:SO_3_Na] ratio were immersed in a dialysis tubing and this dialysis tubing suspended in DPBS buffer under mild agitation. Aliquots of the dialysate were taken at different time points and analysed by HPLC to monitor the release of Pol-B over time (Fig. S5†). Overall PIC particles were able to slow the release of Pol-B, with less than 10% of the antibiotic released after 4 h and 80% released after 48 h. However, these experiments were complicated by two main issues:

(1) There were interactions between Pol-B and the membranes used, as demonstrated by the “slow release” of free Pol-B from the dialysis tubing (Fig. S5†, black squares). Membranes of different molecular weight cut-off (MWCO) were tested to address this limitation, until significant release was observed (over 40% after 8 h) for a 20 KDa MWCO membrane. We believe this “slow release” was mainly the result of self-assembly of Pol-B, forming supramolecular aggregates with bigger hydrodynamic radius than the membrane porosity. Also, some interaction of Pol-B with the membrane was evident for the smaller MWCOs tested, with membranes turning opaque after the experiment.
(2) Under the experimental conditions used, Pol-B is a mixture of species (Fig. S6†), and only one of those (peak at 2.7 min) can be clearly identified in the dialysate.

**Fig. 4:**
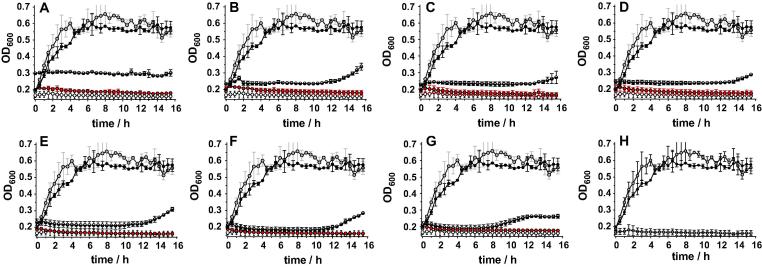
Change in optical density at 600 nm (OD_600_) for *P. aeruginosa* cultures in the absence () and presence of PSS (), Pol-B () and PIC particles () prepared at 0.7 (A), 0.6 (B), 0.5 (C), 0.4 (D), 0.3 (E), 0.2 (F) and 0.1 (G) [N:SO_3_Na] ratio. In each case, the concentration of Pol-B was adjusted to that in the PIC particles employed. Optical density of each of the controls including the media employed (1:1 LB:HEPES, ) is shown for comparison (H). *n* = 3.

We then evaluated the effect of encapsulating Pol-B inside these PIC nanoparticles on the activity of this antimicrobial. We anticipate that upon dilution in media, other electrolytes (*e.g.* NaCl) will screen the electrostatic interactions between Pol-B and PSS, facilitating disruption of the particle and the release of Pol-B. Towards this end, changes in optical density at 600 nm in *P. aeruginosa* cultures were monitored. This is a well-established indirect measurement of microbial growth that correlates any increase in the optical density of the culture to an increase in bacteria numbers [31]. Therefore, the effect PIC particles prepared between 0.7 and 0.1 [N:SO_3_Na] ratios had on the growth of this pathogen was evaluated this way, and compared to the growth in the presence of free Pol-B and free PSS (Fig. 4). While PSS had no effect on the growth of this opportunistic pathogen (Fig. 4A–H, ), with changes in optical density mirroring those for *P. aeruginosa* alone (Fig. 4A–H, ), all of the concentrations of Pol-B tested (122.5–17.5 μg mL^−1^, Fig. 4A–G, ) inhibited *P. aeruginosa*’s growth for at least 16 h. A similar effect on growth could be observed for most of the PIC particles evaluated, with *P. aeruginosa*’s growth inhibited for at least 8 h. The antimicrobial effect of these PIC particles seemed to be formulation dependent, with those incorporating more Pol-B inhibiting the pathogen’s growth for longer (*e.g.* up to 14.5 h for particles prepared at a 0.7 [N:SO_3_Na] ratio).

**Fig. 5:**
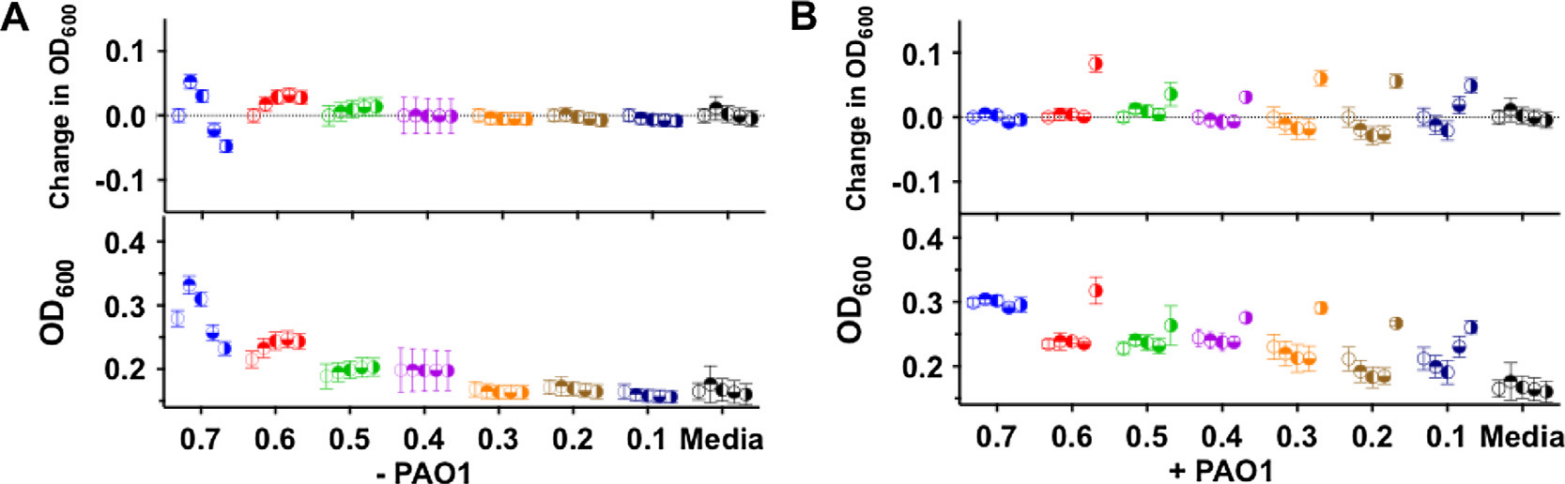
Representative changes in optical density at 600 nm (OD_600_) with time ( 0 h,  2 h,  5 h,  10 h,  15 h) for PIC particles, prepared at different [N:SO_3_Na] ratios, suspended in 1:1 LB:HEPES media in the absence (A) and presence of *P. aeruginosa* (B). Changes in OD_600_ represent the difference between the initial OD_600_ (t = 0 h) and the OD_600_ at each time point. No increase in optical density was observed for any of the individual components in the absence of *P. aeruginosa* (Fig. S7A†). *n* = 3. Each colour represents a different particle formulation.

The lower activity of these PIC nanoparticles compared to free Pol-B was expected, since these nanomaterials should act as reservoirs of Pol-B that is then slowly released from the nanoparticle. More surprising though was the fact that optical density in the presence of the particles was often higher than that in the presence of the same amount of Pol-B ( vs  for each of the particles examined). This difference was more pronounced for those particles containing higher concentrations of Pol-B, with those prepared at a 0.7 [N:SO_3_Na] ratio exhibiting an initial OD_600_ of 0.3, which was maintained through the duration of the experiment. Since these particles were able to scatter light (Fig. 2), and the intensity of this scattering was dependent on the formulation but also the conditions to which these particles were exposed (Fig. 3), we decided to evaluate if these particles could affect the optical density of the media in the absence of any microorganism. Incubation of particles with LB media in the absence of *P. aeruginosa* confirmed that this was the case, with particles increasing the optical density of the media in a formulation dependent fashion (Fig. 5A). No increase in optical density was observed for any of the individual components (Fig. S7A†), suggesting that the observed increase in optical density was a result of the addition of PIC particles. Particles containing more Pol-B led to higher optical densities at 600 nm, with those prepared at a 0.7 [N:SO_3_Na] ratio exhibiting an initial OD_600_ of 0.28 ± 0.01, very similar to that observed in the presence of *P. aeruginosa* (Fig. 5B). Overall, this initial optical density was maintained for the duration of the experiment for most of the PIC particles evaluated, with only small changes in the optical density observed. Only particles prepared at a 0.7 and 0.6 [N:SO_3_Na] ratios exhibited significant changes in OD_600_. Both formulations increased the optical density of the media initially, but particles prepared at 0.7 [N:SO_3_Na] ratio eventually resulted in an overall reduction in optical density, with an OD_600_ of 0.23 after 15 h of incubation (Fig. 5A). This difference in behaviour may reflect the different stabilities of the PIC particles in this media (1:1 LB:HEPES), although no direct correlation should be expected with the stability observed in HEPES/NaCl buffer (Fig. 3).

**Fig. 6:**
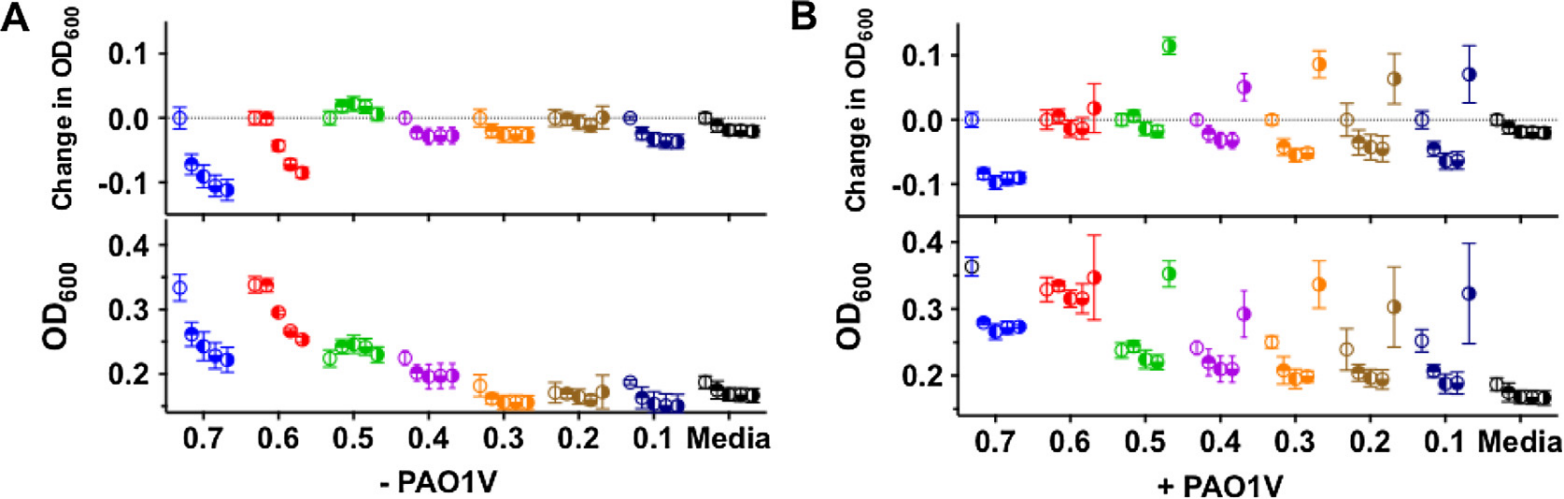
Representative changes in optical density at 600 nm (OD_600_) with time ( 0 h,  2 h,  5 h,  10 h,  15 h) for PIC particles, prepared at different [N:SO_3_Na] ratios, suspended in 1:0.4 LB:HEPES media in the absence (A) and presence of *P. aeruginosa* (B). Changes in OD_600_ represent the difference between the initial OD_600_ (t = 0 h) and the OD_600_ at each time point. No increase in optical density was observed for any of the individual components in the absence of *P. aeruginosa* (Fig. S7B†). *n* = 3. Each colour represents a different particle formulation.

To further investigate the behaviour of the particles in media, a new experiment was set-up in which particles were diluted even more in growth media (1:0.4 LB:HEPES). A similar trend was observed, both in the absence and presence of *P. aeruginosa*, although the higher content of LB media seemed to have a detrimental effect in some of the formulations (Fig. 6A). This effect was more evident for those particles that had a higher effect on the optical density of the media (0.7 and 0.6 [N:SO_3_Na] ratios). As before, in the absence of bacteria, these two formulations increased the optical density of the media, in this case to an OD_600_ of approximately 0.33. However, optical density for these two formulations was then reduced, suggesting some degree of instability under these conditions. As before, inhibition of growth for *P. aeruginosa* in the presence of the particles was formulation dependent, with particles prepared at a 0.7 [N:SO_3_Na] ratio sustaining the longest antimicrobial effect (Fig. 6B and Fig. S8†).

**Fig. 7:**
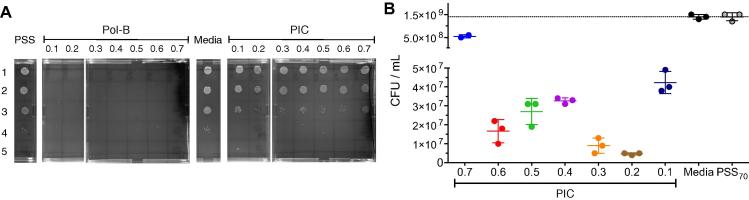
(A) Representative agar plates used to evaluate antimicrobial activity of Pol-B containing PIC particles. Lane 1: No dilution, lane 2: 10^2^-fold dilution, lane 3: 10^4^-fold dilution, lane 4: 10^6^-fold dilution, and lane 5: 10^8^-fold dilution. (B) CFU/mL of *P. aeruginosa* in the absence (media, ) and presence of PSS () or PIC particles prepared at different [N:SO_3_Na] ratios (dots, each colour represents a different particle formulation). Experiments were done in triplicate in 1:1 LB:HEPES media.

Finally, we also investigated the antimicrobial activity of these Pol-B containing particles by incubating *P. aeruginosa* with these particles and counting the resulting colony forming units (CFU). This assay correlates the amount of viable bacteria in a culture to the number of colonies that are formed after spotting an aliquot of this culture on agar plates [31]. Several dilutions were employed to spot the agar plates, to identify suitable conditions for colony counting (Fig. 7A). In agreement with the observed antimicrobial activity during growth measurements using changes in OD_600_, all of the concentrations of free Pol-B tested inhibited the growth of *P. aeruginosa*, and no colonies were observed. Also, the antimicrobial activity of the particles was smaller than that of the free Pol-B (Fig. 7). This effect was formulation dependent but did not correlate with the amount of Pol-B incorporated in each nanoparticle. For instance, particles prepared at a 0.3 [N:SO_3_Na] ratio had a higher activity than those prepared at a 0.4 [N:SO_3_Na] ratio, despite having almost 25% less amount of Pol-B (Table S1†). However, particles prepared at 0.6 [N:SO_3_Na] ratio had a higher activity that those prepared at 0.5 and 0.4 [N:SO_3_Na] ratios, as expected from the amount of Pol-B encapsulated and the stability of the particles formed (Table S1† and Fig. 3). We believe there are several competing mechanisms affecting the observed antimicrobial activities. On the one hand, particle instability will result in “easier” release of Pol-B from unstable particles such as those prepared at 0.7, 0.6 or 0.1 [N:SO_3_Na] ratios. On the other hand, the amount of Pol-B available will then strongly depend on the amount of Pol-B included in the formulation, with those prepared at a 0.1 [N:SO_3_Na] ratio expected to have the lower activity. Overall, all of the concentrations tested (except those prepared at a 0.7 [N:SO_3_Na] ratio, which seems to be an outlier) were able to reduce the number of CFU/mL by approximately 2 orders of magnitude, and a relative reduction of the initial CFUs of over 97%.

## 4 Conclusions

Here we have presented our efforts towards the development of PIC particles for the delivery of Pol-B, an antimicrobial peptide of clinical relevance. Using 70 KDa PSS as the inert component of the formulation, we have identified a range of conditions for which Pol-B containing PIC particles can be prepared. We have evaluated the stability of these particles under simulated physiological conditions (*i.e*. pH, osmotic pressure and temperature). Preliminary evaluation of the antimicrobial activity of these PIC particles has been performed by monitoring their impact on *P. aeruginosa*’s growth. Overall, particles with a higher content of Pol-B can inhibit growth of this pathogen for longer, although this effect was formulation dependent. Finally, we report that, in the absence of bacteria, Pol-B containing particles are able to increase the optical density of the growth media in a particle-dependent fashion, an effect that can reflect the different stabilities of the PIC particles in this medium. Our efforts to optimise antimicrobial activity, including the optimisation of the polymer carrier and the development of degradable and targeted particles will be reported in due course.

## Author contributions

All authors contributed to the experimental set-up and discussed the results. II and FFT designed the nanoparticle synthesis and characterisation, and II, FFT and AMK designed the microbiological assays. II and SM carried out the microbiological assays, and II carried out all other experiments. FFT and AMK secured funding. II and FFT analysed the data and wrote the paper, with all other authors contributing to the final version of the manuscript.
