# Supplementary material for: Preparation and antimicrobial evaluation of polyion complex (PIC) nanoparticles loaded with polymyxin B
Source: Eur Polym J. 2017 Feb;87:478–86. doi: 10.1016/j.eurpolymj.2016.08.023 (PMC5327956; doi:10.1016/j.eurpolymj.2016.08.023)
Supplement: Supplementary data 1 [file mmc1.pdf]

Preparation and antimicrobial evaluation of polyion complex (PIC) nanoparticles loaded with polymyxin B

Electronic Supplementary Information

Ignacio Insua,[a,c] Sieta Majok,[b,c] Anna F. A. Peacock,[a] Anne Marie Krachler,[b,c] and Francisco Fernandez-Trillo.[a,c]

[a] School of Chemistry, [b] School of Biosciences and [c] Institute of Microbiology and Infection, University of Birmingham, B15 2TT Birmingham, UK

Contents

1. PIC particles preparation and characterisation 1
   1.1. TEM micrographs of PIC particles 1
   1.2. Stability of PIC particles under simulated physiological conditions 2
2. Pol-B release from PIC particles 3
3. Antimicrobial activity of PIC particles 4
4. Additional references 4

1. PIC particles preparation and characterisation

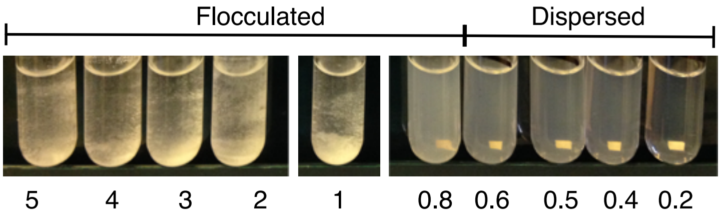

Figure S1 Macroscopic appearance of particles prepared at different [N:SO_3Na] ratios.

Table S1 Hydrodynamic diameter (D_H), ζ -potential and concentration of Pol-B of PIC particles prepared at different [N:SO_3Na] ratios. SD indicates the standard deviation found for the only size or charge population fitted by the software.

[N:SO_3Na] ratio	D_H ± SD (nm)	PdI [a]	ζ -potential ± SD (mV)	[Pol-B] (μM)
**0.7**	186 ± 50	0.07	-29.7 ± 10.2	175
**0.6**	182 ± 43	0.06	-38.0 ± 9.2	150
**0.5**	176 ± 41	0.05	-41.2 ± 9.6	125
**0.4**	174 ± 49	0.08	-41.0 ± 8.2	100
**0.3**	167 ± 37	0.05	-40.1 ± 10.0	75
**0.2**	166 ± 38	0.05	-42.5 ± 7.3	50
**0.1**	169 ± 47	0.08	-41.2 ± 9.2	25

[a] Polydispersity Index (PdI) calculated using the formula: PdI = $(SD/D_H)^2$.[S1]

1.1. TEM micrographs of PIC particles

5 μL of a suspension of PIC particles were deposited on the surface of the TEM grid and it was left to dry at room temperature covered from dust. Once dried, 5 μL of a 1 mg/mL solution of phosphotungstic acid in water were deposited on the grid to stain the nanoparticles for a minute, after which the excess staining solution was removed. Finally, the grid was dried as before.
For the measurement of the average PIC particle diameter, 58 nanoparticles were measured twice (in their longest and shortest diameters), giving an average of 140 nm with a standard deviation of 30 nm from a total of 116 measurements.

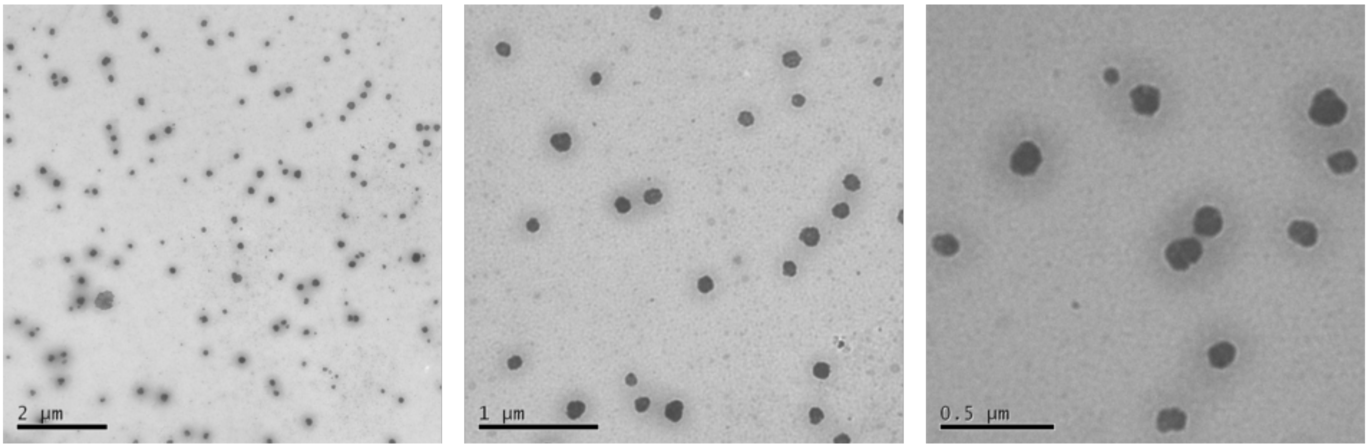

Figure S2 Representative TEM micrographs of PIC prepared at 0.4 [N:SO_3Na] ratio with increasing magnification (left to right).

1.2. Stability of PIC particles under simulated physiological conditions

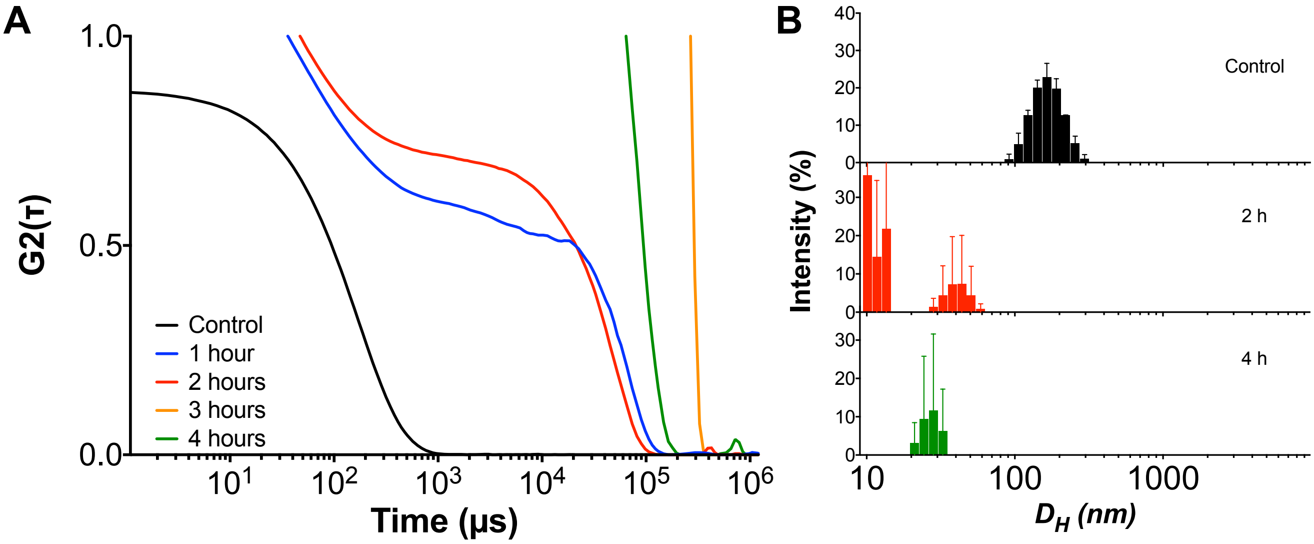

Figure S3 Autocorrelation function (ACF) curves (**A**) and representative size-intensity distributions (**B**) for PIC particles prepared at a 0.1 [N:SO_3Na] ratio in the absence (control) and presence of 154 mM NaCl at 37 °C over time (1-4 hours).

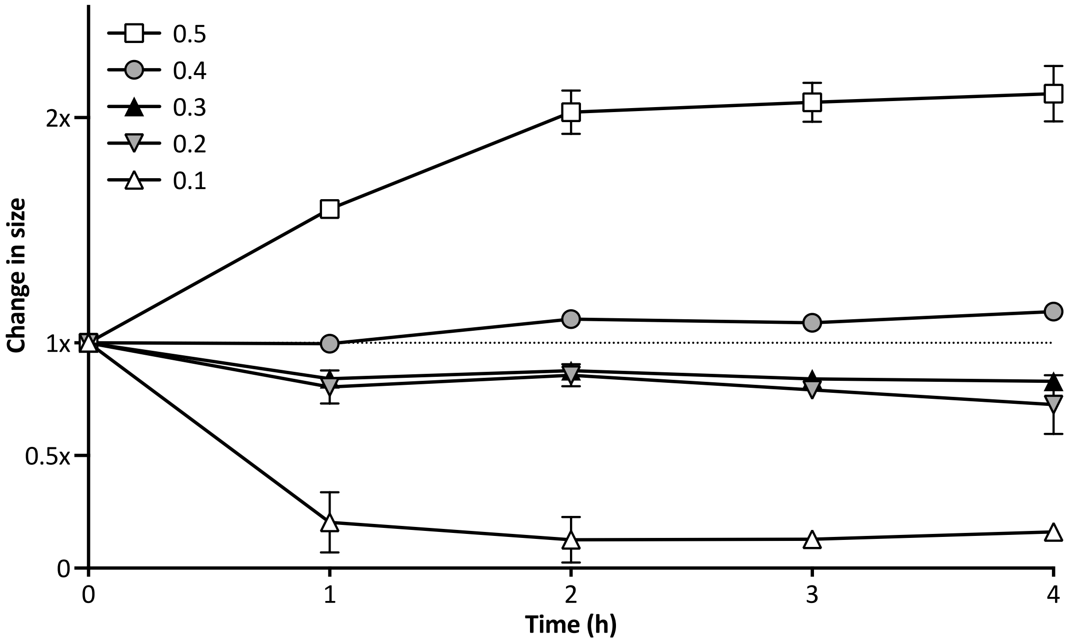

Figure S4 Evolution of PIC particle size (D_H) as measured by DLS when incubated with 154 mM NaCl at 37 °C over time. The size of the nanoparticles was normalised to that of a control in the absence of NaCl (time point 0 h), and was monitored over 4 hours after the addition of salt (time points 1-4 h). Each symbol represents a different [N:SO_3Na] ratio.[‡] n = 3.

2. Pol-B release from PIC particles

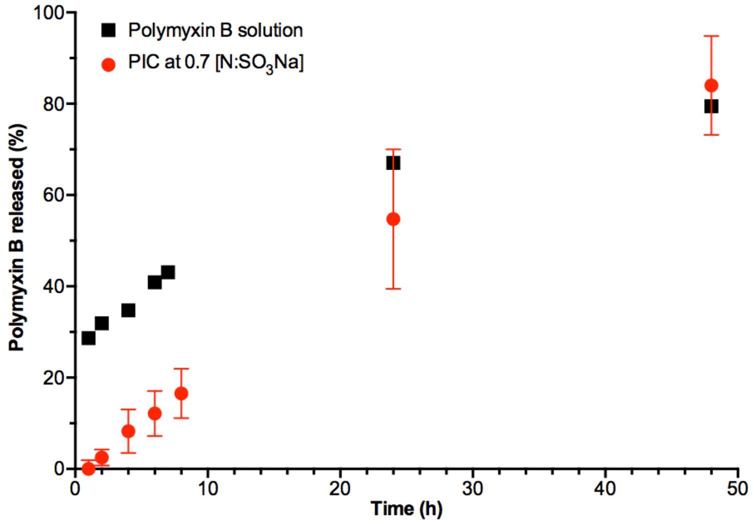

Figure S5: Pol-B content found in the dialysate of PIC particles prepared at 0.7 [N:SO_3Na] ratio (red circles) over time. Content was normalised to that found in a 175 μM Pol-B sample (drug loading in these PIC particles) diluted down to the total dialysis volume (100%) and compared to the rate of release for a Pol-B solution at the same concentration (black squares). n = 3.

[‡] PIC particles at 0.7 and 0.6 [N:SO_3H] have not been included since when these formulations were exposed to NaCl, they swelled to give more than one size distributions as indicated in **Figure 3.**

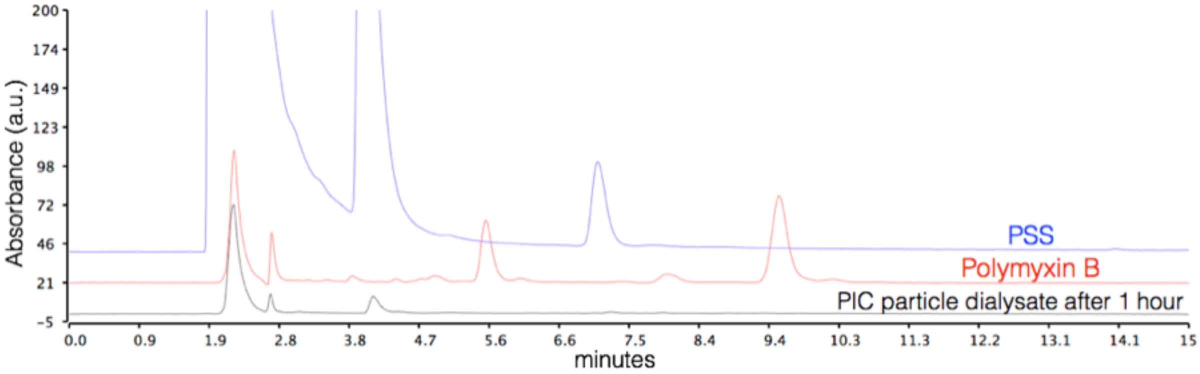

Figure S6: Representative RP-HPLC chromatograms of 70 KDa PSS (top), Pol-B (middle) and PIC particles prepared at a 0.7 [N:SO_3Na] ratio (bottom). Peak at *R*t = 2.7 min was used to monitor the release of Pol-B.

3. Antimicrobial activity of PIC particles

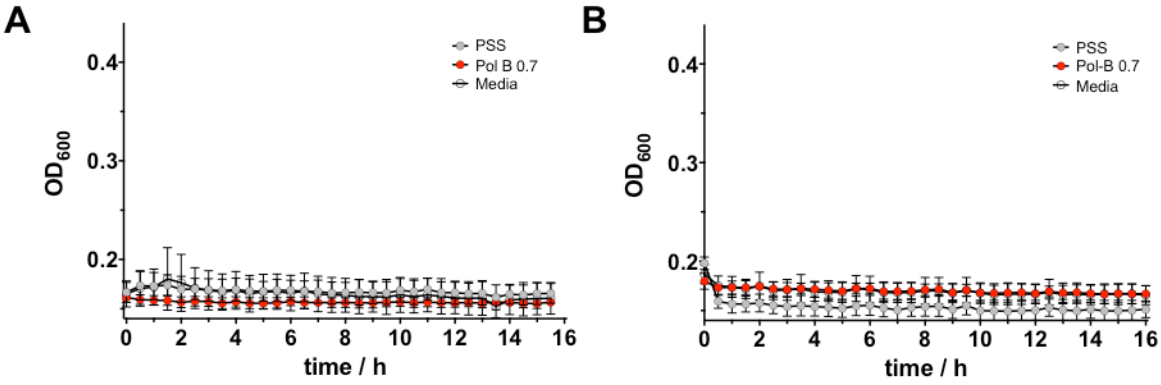

Figure S7 Change in optical density at 600 nm (OD_{600}) for media A) 1:1 LB:HEPES and B) 1:0.4 LB:HEPES; in the absence (○) and presence of PSS (●) and Pol-B (●).

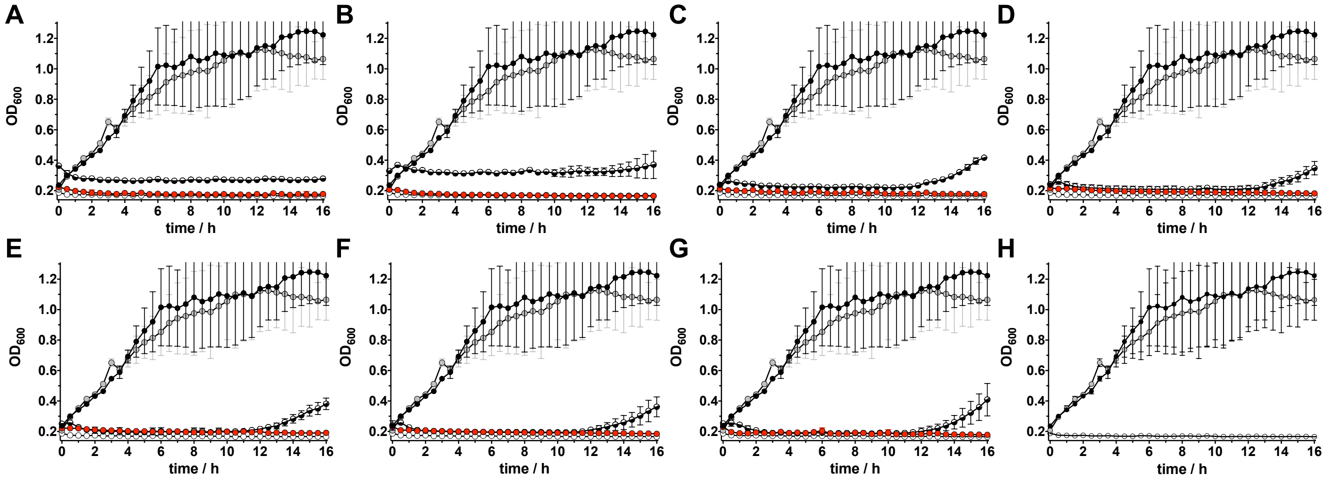

Figure S8 Change in optical density at 600 nm (OD_{600}) for *P. aeruginosa* cultures in the absence (●) and presence of PSS (●), Pol-B (●) and PIC particles (◒) prepared at 0.7 (**A**), 0.6 (**B**), 0.5 (**C**), 0.4 (**D**), 0.3 (**E**), 0.2 (**F**) and 0.1 (**G**) [N:SO_3Na] ratio. In each case, the concentration of Pol-B was adjusted to that in the PIC particles employed. Optical density of each of the controls including the media employed (1:0.4 LB:HEPES, ○) is shown for comparison (**H**). $n = 3$.

4. Additional references

[S1] Measuring the size of nanoparticles in aqueous media using batch-mode Dynamic Light Scattering, 1st ed., NIST, U.S. Department of Commerce, 2015. doi:10.6028/nist.sp.1200-6.
